# Complete Genome Sequence of a Virulent Leptospira interrogans Serovar Copenhageni Strain, Assembled with a Combination of Nanopore and Illumina Reads

**DOI:** 10.1128/MRA.00200-20

**Published:** 2020-04-23

**Authors:** Alejandro Llanes, Dhani Prakoso, Carlos Mario Restrepo, Sreekumari Rajeev

**Affiliations:** a Centro de Biología Celular y Molecular de Enfermedades, Instituto de Investigaciones Científicas y Servicios de Alta Tecnología, Panama City, Panama; b College of Veterinary Medicine, University of Florida, Gainesville, Florida, USA; Indiana University, Bloomington

## Abstract

Here, we present the complete genome sequence of a highly virulent Leptospira interrogans serovar Copenhageni strain isolated from a dog with severe leptospirosis. In this work, a gapless genome draft was assembled with a combination of Nanopore and Illumina data of relatively low coverage.

## ANNOUNCEMENT

*Leptospira* species are spirochete bacteria that colonize proximal renal tubules of mammals, and infections may cause fatal disease in humans and animals. In this study, we assembled the genome of Leptospira interrogans strain SK1 and compared it with those of L. interrogans serovar Copenhageni (strain Fiocruz L1-130) (1) and L. interrogans serovar Lai (strain 56601) (2). The SK1 strain was isolated from the blood of a canine patient that succumbed to severe clinical leptospirosis (3). The strain was isolated through routine *Leptospira* culture protocols using Ellinghausen-McCullough-Johnson-Harris medium, as described previously (1).

Genomic DNA from strain SK1 was isolated with the MasterPure complete DNA and RNA purification kit (Epicentre, USA). Sequencing libraries for the MiSeq and MinION platforms were prepared with the Nextera XT DNA library preparation kit (Illumina, USA) and the ligation sequencing kit (SQK-LSK109; Oxford Nanopore Technologies, UK), respectively, following the manufacturers’ instructions. Quality control was performed with FastQC (https://www.bioinformatics.babraham.ac.uk/projects/fastqc). In the case of Nanopore reads, we used Guppy v.3.2.4 for base calling and Porechop v.0.2.4 (https://github.com/rrwick/Porechop) for adapter trimming. *De novo* assembly was performed with Canu v.2.0 (4) using only the Nanopore reads. The Canu assembly was initially polished with Medaka v.0.11.4 (https://nanoporetech.github.io/medaka) using probabilistic model r941_min_fast_g303. Pilon v.1.23 (5) was used for hybrid polishing with the Illumina reads. To estimate the number of sequencing errors after each polishing step, Illumina reads were aligned to the Nanopore assembly with BWA v.0.7.12 (6). BCFtools v.1.9 (7) was then used to call for single-nucleotide variants and insertion/deletions (indels), which were considered to be errors if their mapping quality score was above 20. RATT was used to transfer the genes annotated in the Fiocruz L1-130 genome to the SK1 assembly. Prokka v.1.14.3 (8) was used to complement the RATT results. Default parameters were used for all software except where otherwise noted.

Illumina and Nanopore sequencing generated 1.1 million 300-bp paired-end reads and 142,300 reads with a median length of 8 kb, respectively. *De novo* assembly of Nanopore reads resulted in 2 contigs, consistent with the 2 *Leptospira* chromosomes. The number of suspected errors decreased from 14,672 (0.3%) in the original Canu assembly to 92 (0.002%) after polishing with Pilon. Of these, only 6 errors seemed to affect putative coding sequences and were corrected manually.

**Fig 1:**
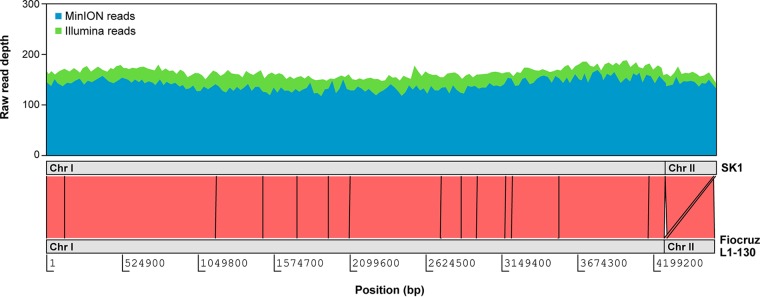
Comparison of the genomes of L. interrogans serovar Copenhageni strains SK1 and Fiocruz L1-130. Pink bands represent regions of shared sequence similarity. Coverage of Nanopore and Illumina reads is plotted along the SK1 genome, averaged over a 500-bp sliding window. Chr, chromosome.

The SK1 genome is very similar to that of strain Fiocruz L1-130 in terms of synteny (Fig. 1) and global metrics, including a total size of 4,630,180 bp, a G+C content of 35%, and 3,731 protein-coding genes. However, at least 11 pseudogenes from Fiocruz L1-130 appear to have functional orthologs in the genomes of strains SK1 and 56601, despite the latter belonging to L. interrogans serovar Lai. Conversely, genes *folA* and *folC*, encoding dihydrofolate reductase and dihydrofolate synthase, respectively, appear to have become pseudogenes in SK1, which suggests impaired folate metabolism in this strain.

### Data availability.

The genome sequence of the SK1 strain and associated data were deposited under BioProject number PRJNA600490, GenBank accession numbers CP048830 (chromosome I) and CP048831 (chromosome II), and SRA accession numbers SRX7657977 (Illumina reads) and SRX7730232 (MinION reads).

## References

[1] Nascimento A, Ko AI, Martins EAL, Monteiro-Vitorello CB, Ho PL, Haake DA, Verjovski-Almeida S, Hartskeerl RA, Marques MV, Oliveira MC, Menck CFM, Leite LCC, Carrer H, Coutinho LL, Degrave WM, Dellagostin OA, El-Dorry H, Ferro S, Ferro MIT, Furlan LR, Gamberini M, Giglioti EA, Góes-Neto A, Goldman GH, Goldman MHS, Harakava R, Jerônimo SMB, Junqueira-de-Azevedo ILM, Kimura ET, Kuramae EE, Lemos EGM, Lemos MVF, Marino CL, Nunes LR, De Oliveira RC, Pereira GG, Reis MS, Schriefer A, Siqueira WJ, Sommer P, Tsai SM, Simpson AJG, Ferro JA, Camargo LEA, Kitajima JP, Setubal JC, Van Sluys MA 2004 Comparative genomics of two *Leptospira interrogans* serovars reveals novel insights into physiology and pathogenesis. J Bacteriol 186:2164–2172. doi:10.1128/jb.186.7.2164-2172.2004. 15028702 PMC374407

[2] Ren SX, Fu G, Jiang XG, Zeng R, Miao YG, Xu H, Zhang YX, Xiong H, Lu G, Lu LF, Jiang HQ, Jia J, Tu Y-F, Jiang J-X, Gu W-Y, Zhang Y-Q, Cai Z, Sheng H-H, Yin H-F, Zhang Y, Zhu G-F, Wan M, Huang H-L, Qian Z, Wang S-Y, Ma W, Yao Z-J, Shen Y, Qiang B-Q, Xia Q-C, Guo X-K, Danchin A, Saint Girons I, Somerville RL, Wen Y-M, Shi M-H, Chen Z, Xu J-G, Zhao G-P 2003 Unique physiological and pathogenic features of *Leptospira interrogans* revealed by whole-genome sequencing. Nature 422:888–893. doi:10.1038/nature01597. 12712204

[3] Larson CR, Dennis M, Nair RV, Llanes A, Peda A, Welcome S, Rajeev S 2017 Isolation and characterization of *Leptospira interrogans* serovar Copenhageni from a dog from Saint Kitts. JMM Case Rep 4:e005120. doi:10.1099/jmmcr.0.005120. 29188067 PMC5692236

[4] Koren S, Walenz BP, Berlin K, Miller JR, Bergman NH, Phillippy AM 2017 Canu: scalable and accurate long-read assembly via adaptive *k*-mer weighting and repeat separation. Genome Res 27:722–736. doi:10.1101/gr.215087.116. 28298431 PMC5411767

[5] Walker BJ, Abeel T, Shea T, Priest M, Abouelliel A, Sakthikumar S, Cuomo CA, Zeng Q, Wortman J, Young SK, Earl AM 2014 Pilon: an integrated tool for comprehensive microbial variant detection and genome assembly improvement. PLoS One 9:e112963. doi:10.1371/journal.pone.0112963. 25409509 PMC4237348

[6] Li H, Durbin R 2010 Fast and accurate long-read alignment with Burrows-Wheeler transform. Bioinformatics 26:589–595. doi:10.1093/bioinformatics/btp698. 20080505 PMC2828108

[7] Li H 2011 A statistical framework for SNP calling, mutation discovery, association mapping and population genetical parameter estimation from sequencing data. Bioinformatics 27:2987–2993. doi:10.1093/bioinformatics/btr509. 21903627 PMC3198575

[8] Seemann T 2014 Prokka: rapid prokaryotic genome annotation. Bioinformatics 30:2068–2069. doi:10.1093/bioinformatics/btu153. 24642063

